# Supplementary material for: The genome sequence of the solitary wasp, Crossocerus cetratus (Shuckard, 1837) (Hymenoptera: Crabronidae)
Source: Wellcome Open Res. 2026 Jun 13;11:363. [Version 1] doi: 10.12688/wellcomeopenres.26724.1 (PMC13639349; doi:10.12688/wellcomeopenres.26724.1)
Supplement: Peer review history

# Reviewer report: Reviewer response for version 1

**DOI:** 10.21956/wellcomeopenres.29426.r168194

**Published:** 2026-10-05

**Authors:** Angelica Penteado Dias

**Affiliations:** 1 Universidade Federal de São Carlos – UFSCar, São Carlos, SP, Brazil

The article refers to genome assembly from an individual female *Crossocerus cetratus* (solitary wasp; Arthropoda; Insecta; Hymenoptera; Crabronidae). The genome sequence and the mitochondrial genome has been assembled. This assembly was generated as part of the Darwin Tree of Life project, which produces genomes for eukaryotic species found in Britain and Ireland.The text is clear, and all procedures are grounded in up-to-date, explanatory bibliographic references. It serves as an excellent reference for understanding and studying Hymenoptera, acting as a model for future studies.

Are sufficient details of methods and materials provided to allow replication by others?

Yes

Is the rationale for creating the dataset(s) clearly described?

Yes

Are the datasets clearly presented in a useable and accessible format?

Yes

Are the protocols appropriate and is the work technically sound?

Yes

Reviewer Expertise:

Hymenoptera taxonomy

I confirm that I have read this submission and believe that I have an appropriate level of expertise to confirm that it is of an acceptable scientific standard.

# Reviewer report: Reviewer response for version 1

**DOI:** 10.21956/wellcomeopenres.29426.r159445

**Published:** 2026-07-01

**Authors:** Kay Lucek

**Affiliations:** 1 University of Neuchâtel, Neuchâtel, Switzerland

The authors present the chromosome level genome assembly of a female specimen of the solitary wasp *Crossocerus cetratus*. The assembly consists of 10 chromosomes and is not annotated for genes. The assembly is highly complete as revealed by the high BUSCO score and the Earth Biogenome Project summary metrics. Sequencing and genome assembly follow the current state of the art and use established methods. The assembly has been generated using the established pipelines of the Darwin Tree of Life Consortium.

 The methods section is very detailed, making the assembly reproducible. For this species, RNA has been sequenced and made available. However, the sex of the specimen used for this dataset is not indicated. Also, gene annotation will be outsourced to ENA, which is a bit surprising as all data is already at hand.

 The biological background seems adequate given that there is not much knowledge about this species. The provided reference genome will provide an invaluable resource for future research on this species and solitary wasps in general.

Are sufficient details of methods and materials provided to allow replication by others?

Yes

Is the rationale for creating the dataset(s) clearly described?

Yes

Are the datasets clearly presented in a useable and accessible format?

Yes

Are the protocols appropriate and is the work technically sound?

Yes

Reviewer Expertise:

Biodiversity genomics

I confirm that I have read this submission and believe that I have an appropriate level of expertise to confirm that it is of an acceptable scientific standard.
